# Alleviation of senescence and epithelial-mesenchymal transition in aging kidney by short-term caloric restriction and caloric restriction mimetics via modulation of AMPK/mTOR signaling

**DOI:** 10.18632/oncotarget.14884

**Published:** 2017-01-28

**Authors:** Dan Dong, Guang-yan Cai, Yi-chun Ning, Jing-chao Wang, Yang Lv, Quan Hong, Shao-yuan Cui, Bo Fu, Ya-nan Guo, Xiang-mei Chen

**Affiliations:** ^1^ Department of Nephrology, Chinese PLA General Hospital, Chinese PLA Institute of Nephrology, State Key Laboratory of Kidney Diseases, National Clinical Research Center of Kidney Diseases, Beijing, P. R. China; ^2^ Department of Nephrology, First Hospital of Jilin University, Changchun, Jilin, P. R. China

**Keywords:** senescence, epithelial-mesenchymal transition, short-term caloric restriction, caloric restriction mimetics, AMPK/mTOR signaling, Gerotarget

## Abstract

Renal fibrosis contributes to declining renal function in the elderly. What is unclear however, is whether epithelial-mesenchymal transition (EMT) contributes to this age-related renal fibrosis. Here, we analyzed indicators of EMT during kidney aging and investigated the protective effects and mechanisms of short-term regimens of caloric restriction (CR) or caloric restriction mimetics (CRMs), including resveratrol and metformin. High glucose was used to induce premature senescence and EMT in human primary proximal tubular cells (PTCs) *in vitro*. To test the role of AMPK-mTOR signaling, siRNA was used to deplete AMPK. Cellular senescence and AMPK-mTOR signaling markers associated with EMT were detected. CR or CRMs treatment alleviated age-related EMT in aging kidneys, which was accompanied by activation of AMPK-mTOR signaling. High glucose induced premature senescence and EMT in PTCs *in vitro*, which was accompanied by down-regulation of AMPK/mTOR signaling. CRMs alleviated high glucose-induced senescence and EMT via stimulation of AMPK/mTOR signaling. Activation of AMPK/mTOR signaling protected PTCs from high glucose-induced EMT and cellular senescence. Short-term regimens of CR and CRMs alleviated age-related EMT via AMPK-mTOR signaling, suggesting a potential approach to reducing renal fibrosis during aging.

## INTRODUCTION

According to the data from the United States Renal Data System, the number of patients on maintenance dialysis will double over the next few years, and a relatively large number of the patients newly diagnosed with chronic kidney disease (CKD) each year are elderly [1]. CKD, which is defined by reduced glomerular filtration rate, proteinuria, or structural kidney disease, is a growing problem among the aging population, to the extent that the elderly have an average prevalence of CKD that is three to five times higher than that observed in young and middle-aged populations [2]. Accordingly, CKD predisposes the elderly to a high risk of cardiovascular events and premature death.

Morphological and functional changes that accompany kidney aging are thought to contribute the development of CKD in the elderly. For example, processes that characterize kidney aging include glomerulosclerosis, interstitial fibrosis, tubular atrophy, vascular sclerosis, and loss of renal function [3]. The mechanistic basis of kidney aging is cellular senescence, which is characterized by the inability of cells to proliferate despite the presence of ample space, nutrients and growth factors in the medium [4, 5]. Although renal fibrosis has been observed in elderly individuals in the absence of overt CKD [6], the relationship between cellular senescence and fibrosis during kidney aging is yet to be determined.

EMT is the process whereby differentiated epithelial cells undergo a phenotypic conversion that gives rise to matrix-producing fibroblasts and myofibroblasts. EMT is increasingly recognized as a key process that contributes to kidney fibrosis and the decline of renal function [7]. Age-related changes in the levels of transforming growth factor-β (TGF-β), epidermal growth factor (EGF), insulin-like growth factor-1 (IGF-1) and vascular endothelial growth factor (VEGF) result in a complex shift of the microenvironmental milieu that is thought to affect tissue homeostasis under both normal and abnormal conditions, triggering EMT and progressive fibrosis [8]. Given that senescence and EMT play well-documented roles in the etiology and progression of age-associated CKD, the development of therapeutic interventions to retard or reverse these processes is warranted.

Caloric restriction has a variety of beneficial effects on health, including the prolongation of lifespan. We have previously demonstrated that short-term (8 week) CR reduced the rate of renal senescence in rat by increasing autophagy and subsequently reducing oxidative damage [9]. Consistent with this, the CRM metformin has been shown to oppose aging in SHR mice via a mechanism involving transcriptional repression of genes encoding key drivers of the EMT program [10]. Similarly, metformin has been shown to impede TGF-β-promoted loss of the epithelial marker E-cadherin in MCF-7 breast cancer cells (11), and to prevent accumulation of the mesenchymal marker vimentin in Madin-Darby canine kidney cells [11, 12]. Similar results have been reported for another CRM, the stilbene derivative resveratrol, which has been shown to inhibit senescence in endothelial progenitor cells [13] and WI-38 fibroblasts [14], and to reduce epidermal growth factor-induced EMT in MCF-7 cells [15].

These observations notwithstanding, the effects of short-term regimens of CR and CRM on cellular senescence and EMT in the aging kidney remain unknown. Here, we asked whether short-term CR and CRM treatment could alleviate renal cell senescence and EMT in the aging kidney, and evaluated the mechanistic basis for these effects. Our study provides a rationale for the development of EMT- and cellular senescence-based therapeutics for the protection of the kidney, and for the treatment of CKD in the elderly population.

## RESULTS

### Age-related metabolic parameters and renal functions in the YAL, OAL, OCR and OMET groups of SD rats

Body weight, 24 h urine protein (PRO/24 h), triglyceride (TG) and cholesterol (CH) were increased in the OAL group compared to the YAL group. After CR or treatment with metformin for 8 weeks, body weight, PRO/24 h and TG were decreased in both the OCR and OMET groups compared to the OAL group (Table 1).

**Table 1:** Age-related metabolic parameters and renal functions in four groups of rats

	YAL (n= 6)	OAL (n= 8)	OCR (n= 8)	OMET (n= 8)
Body Weight (g)	162.17±7.78^a^	715.32±121.16	588.33±97.94^a,b^	671.8±78.05^a,b^
PRO/24 h (mg)	1.94±1.07^a^	30.16±4.58	14.69±3.42^a,b^	21.31±3.46^a,b^
Scr (μmol/L)	18.32±1.94	18.93±0.14	18.81±0.07	18.29±0.46
BUN (mmol/L)	5.28±0.73	5.16±0.3	5.47±0.12	5.54±0.28^b^
TG (mmol/L)	0.47±0.18^a^	1.56±0.43	0.67±0.24^a,b^	0.97±0.28^a^
CH (mmol/L)	1.4±0.47^a^	2.3±0.62	2.27±0.51^b^	2.26±0.82^b^
ALB (g/L)	39.78±1.5	35.52±2.65	37.03±1.29	35.22±4.97
TP (g/L)	61.65±1.74	66.3±4.34	66±1.8	64.12±6.61

The data are presented as means ± SD, ^a^p < 0.05 YAL, OCR, OMET vs. OAL, ^b^p < 0.05 OCR, OMET vs. YAL, ^&^p < 0.05 OMET vs. OCR

### CR and metformin treatment alleviated age-related EMT in aging kidneys

**Figure 1:**
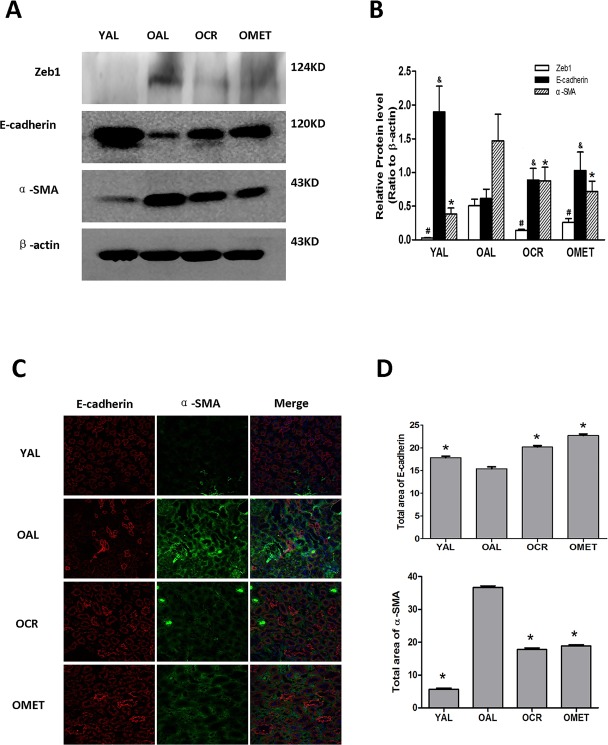
The effect of short-term CR and metformin treatment on EMT in the kidneys of aging rats. After CR or metformin treatment for 8 weeks **A**. the kidney proteins were extracted and blotted with antibodies against Zeb1, E-cadherin, α-SMA. β-actin was used to equalize the load amounts. **B**. The values from the western blots are expressed as means ± SD of each group relative to β-actin. ^#, &^,*P < 0.05 vs. OAL. **C**. Frozen sections of rat kidney were labeled for E-cadherin (red) and α-SMA (green). The nuclei were counterstained with DAPI (blue). A representative image for each group is shown. Magnification, ×400. **D**. The values shown are areas for the different colors of each group and are expressed as means ± SD, *P < 0.05 vs. OAL.

Renal expression of α-smooth muscle actin (α-SMA), a specific marker of mesenchymal fibroblasts, and the transcription factor Zeb1, an inducer of EMT [16] were higher in the OAL group than in the YAL group. In contrast, expression of the epithelial marker E-cadherin were lower in the OAL group than in the YAL group (Figure 1). After short-term CR or metformin treatment, renal expression of both Zeb1 and α-SMA was lower in the OCR and OMET groups than in the OAL group, whereas renal expression of E-cadherin showed the opposite trend (Figure 1). These results indicate that EMT was more characteristic of the kidneys of the older rats than the younger rats, and that both CR and metformin treatment mitigated against EMT in the kidneys of the older rats.

### CR and metformin treatment alleviated age-related renal cell senescence in aging kidneys

**Figure 2:**
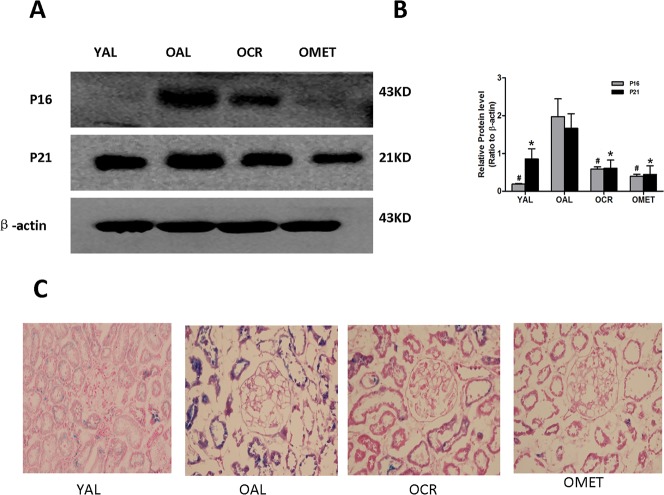
Effect of short-term CR and metformin treatment on cellular senescence in the kidneys of old rats **A**. Protein was extracted and subjected to western blotting with antibodies against P16, P21 or β-actin. **B**. The bottom panel shows quantitation of the western blot results. The values are expressed as means ± SD of each group relative to β-actin. ^#^, *P < 0.05 vs. OAL. **C**. SA-β-gal results for renal tissues from the YAL, OAL, OCR and OMET groups. Magnification, ×400. Blue precipitates in the cytoplasm were observed in the senescent cells.

A characteristic feature of cell senescence is cell cycle arrest, which is indicated by changes in cellular levels of the telomeres, P16 and P21 [17]. As such, we next compared the renal expression levels of P16 and P21 in the YAL, OAL, OCR and OMET groups of rats. Expression of P16 and P21 was increased in the OAL group, compared with the YAL group. However, expression of P16 and P21 was lower in the OCR and OMET groups than in the OAL group (Figure 2A & 2B). Similar results were obtained with respect to SA-β-gal (Figure 2C), the activity of which has been previously shown to be elevated in senescent cells and tissues [18]. Our results indicated that CR and metformin treatment alleviated cellular senescence in rat kidney.

### CR and metformin treatment activated AMPK-mTOR signaling

**Figure 3:**
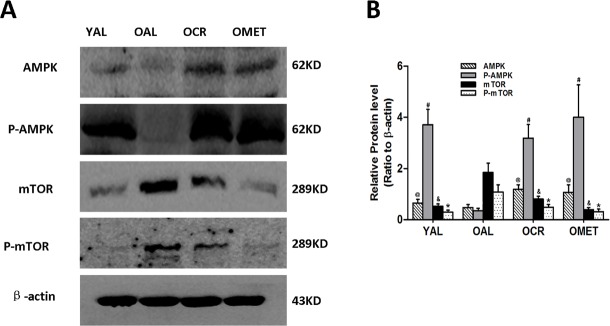
Effect of short-term CR and metformin treatment on AMPK/mTOR signaling in the kidneys of old rats **A**. At least 50 μg kidney lysates were immunoblotted with anti-AMPK, anti-P-AMPK, anti-mTOR and anti-P-AMPK antibodies as described in Western blotting. β-actin was used to equalize the load amounts. **B**. The bottom panel shows the quantitation of the Western blot results. The values are expressed as means ± SD of each group relative to β-actin. ^@, #, &,^ *P < 0.05 vs. OAL.

Western blot analysis indicated that AMPK/mTOR signaling was lower in the OAL group than in the YAL group, and that CR and metformin treatment activated this pathway in the OCR and OMET groups (Figure 3).

### High glucose-induced senescence and EMT was accompanied by down-regulation of AMPK/mTOR signaling in PTCs

**Figure 4:**
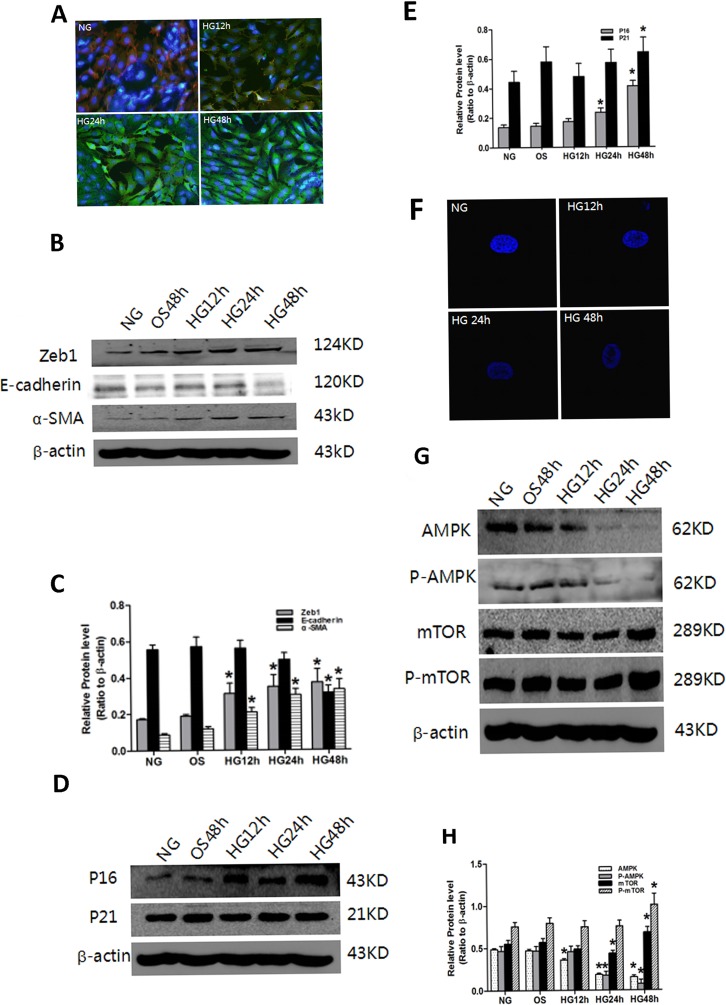
Effect of HG on senescence, EMT and AMPK/mTOR signaling in PTC PTC were exposed to HG (30 mM D-glucose) for 0–48 h. **A**. Decreased levels of the epithelial cell marker E-cadherin (Red), increased levels of the mesenchymal cell markers α-SMA (Green) and nucleus (Blue) were measured by confocal microscopy. Magnification, ×400. F: The PTC were stained with DAPI, SAHF are preferentially detected in HG-induced cells. Numbers indicate percentage of SAHF-positive cells (means ± s.e.m.); n = 3. and heterochromatin foci were observed in the senescent cells. Magnification, ×600. Protein was extracted and blotted with antibodies against **B**. the EMT markers Zeb1, E-cadherin and α-SMA, **D**. the senescence markers P16 and P21, **G**. the signaling proteins AMPK, P-AMPK, mTOR and P-mTOR, or β-actin. Each example shown is representative of three independent experiments. **C**., **E**., **F**. The values are expressed as means ± SD of three experiments for each condition determined from densitometry relative to β-actin. *p < 0.05 vs. control. OS indicates that the cells were cultured in 5.5 mM D-glucose plus 24.5 mM mannitol for 48 h. There was no significant difference between the OS and NG conditions (p > 0.05).

We next sought to elucidate the mechanistic basis of the induction of senescence and EMT in high glucose-treated PTCs. Induction of P16, P21, Zeb1 and α-SMA, repression of E-cadherin and AMPK/mTOR signaling, and increased SAHF were observed in PTCs when treated with high glucose, but not with the osmolarity control OS (Figure 4B-4E). Comparable results with respect to α-SMA and E-cadherin were obtained with immunofluorescence staining, which also demonstrated epithelial cell to mesenchymal morphologic transformation of PTCs (Figure 4A).

### Metformin and resveratrol alleviated high glucose-induced senescence and EMT via stimulation of AMPK/mTOR signaling

**Figure 5:**
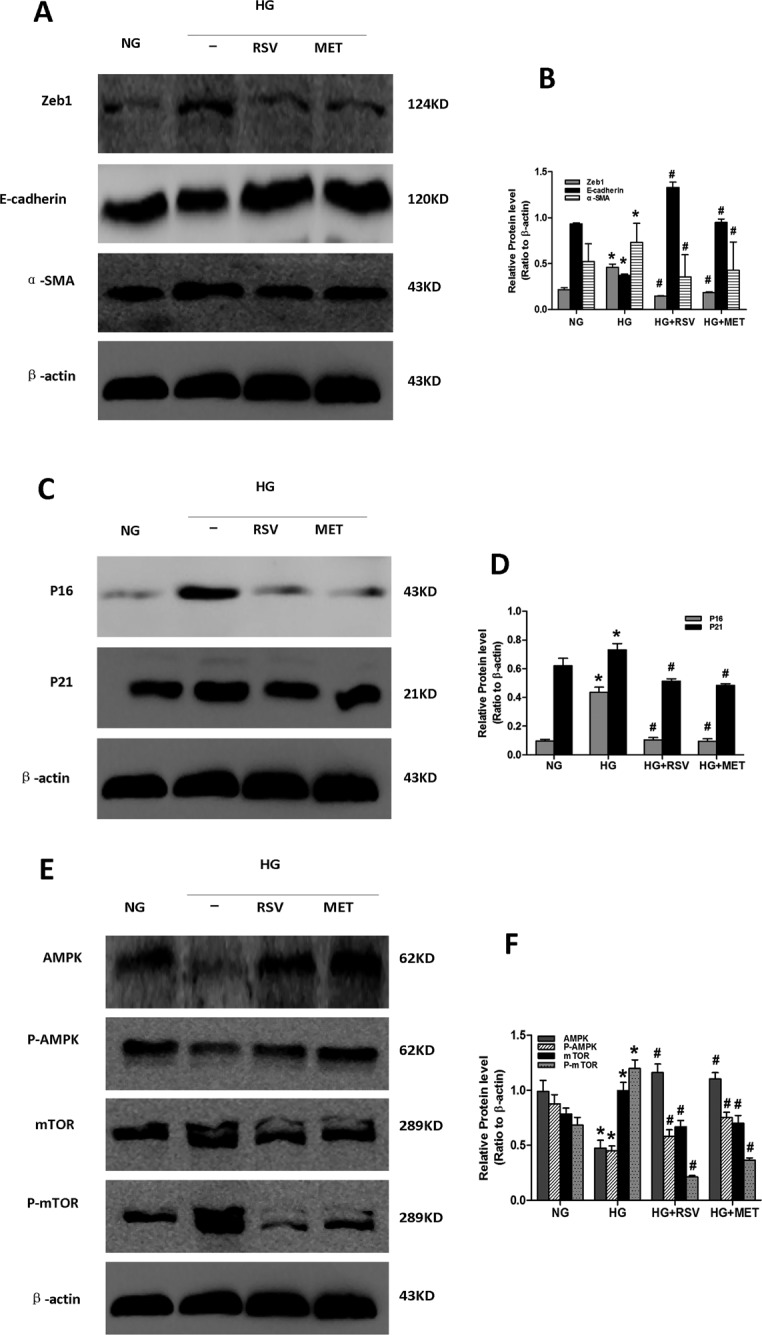
Effect of resveratrol and metformin on HG-induced senescence, EMT and AMPK/mTOR signaling Cells incubated in the following conditions for 48 h were harvested for western blot analysis. NG: NG (5.5 mM) treatment. HG: HG (30 mM) treatment. HG+RSV: HG (30 mM) and Resveratrol (50 μmol/L) treatment. HG+MET: HG (30 mM) and Metformin (2 mmol/L) treatment. At least 50 μg of cell lysates were immunoblotted with antibodies against specific protein markers for **A**. EMT, **C**. senescence and **E**. AMPK/mTOR signaling or β-actin. β-actin was used to equalize the load amounts. **B**., **D**., **F**. The bottom panels show the comparisons of the protein level among the groups as ratio to β-actin. Each example shown is representative of three independent experiments. The values are means ± SD determined from densitometry relative to β-actin for 3 experiments for each condition. *p < 0.05 vs. NG. ^#^P < 0.05 vs. HG alone.

Induction of E-cadherin expression and AMPK/mTOR signaling, and repression of P16, P21, Zeb1 and α-SMA expression, were observed in the high glucose plus CRM (metformin or resveratrol) co-stimulated cells compared to cells stimulated with HG alone (Figure 5). These results supported the hypothesis that metformin and resveratrol can alleviate high glucose-induced senescence and EMT via stimulation of AMPK/mTOR signaling. Thus, the AMPK/mTOR pathway appears to play a key role in the regulation of senescence and EMT of the aging kidney.

### Activation of AMPK/mTOR signaling protected PTCs from high glucose-induced EMT and cellular senescence

**Figure 6:**
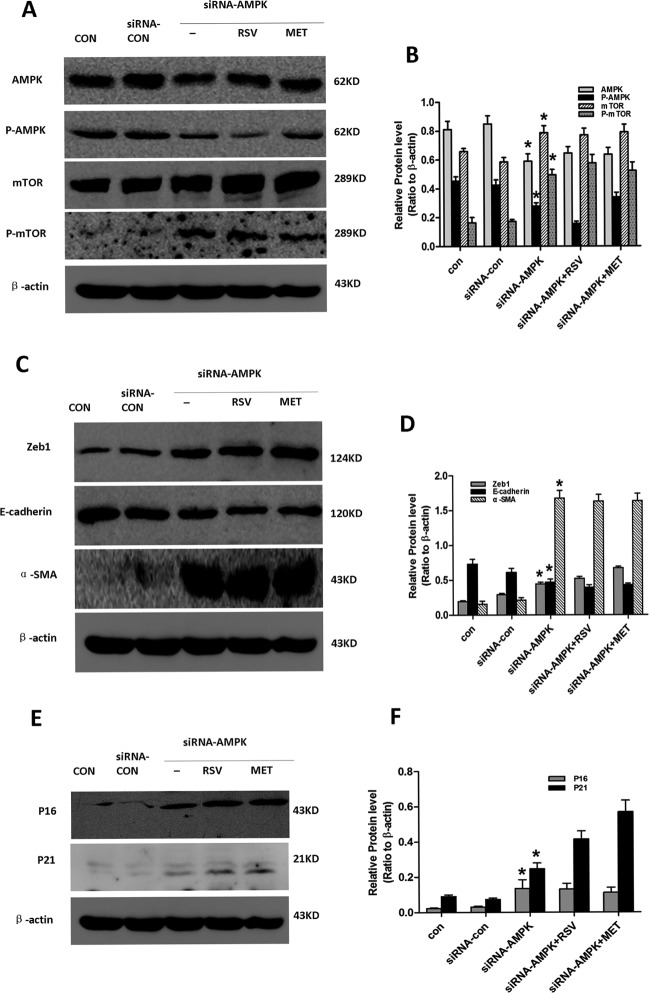
Effect of resveratrol and metformin on silenced AMPK-induced senescence, EMT Cells in the Con, siRNA-con, siRNA-AMPK, siRNA-AMPK+RSV and siRNA-AMPK+MET groups were cultured in normal glucose for 24 h, after which siRNA-AMPK+RSV cells were treated with 50μM resveratrol, and siRNA-AMPK+MET cells were treated with 2mM metformin. Subsequently, all cells were incubated for another 24h. Cells were then harvested for western blot analysis with antibodies against **A**. AMPK, P-AMPK, mTOR, P-mTOR, **C**. Zeb1, E-cadherin, α-SMA, and **E**. P16, P21 or β-actin. β-actin was used to equalize the load amounts. Each example shown is representative of three independent experiments. **B**., **D**., **F**. Values are means ± SD of 3 experiments for each condition determined from densitometry relative to β-actin. *p < 0.05 vs. con.

We next used AMPK siRNA to confirm the role of AMPK/mTOR signaling in cellular senescence and EMT (Figure 6). Compared with control cells, reduced AMPK/mTOR signaling and E-cadherin expression, and increased expression of Zeb1, α-SMA, P16 and P21 were observed in AMPK-silenced PTCs, but not in metformin- or resveratrol- treated AMPK-silenced PTCs. These results indicate that metformin and resveratrol alleviate EMT and senescence via AMPK/mTOR signaling, and that activation of AMPK/mTOR protected PTCs from high glucose-induced EMT and cellular senescence.

## DISCUSSION

Decades of research have associated age-related loss of kidney function with increased renal vascular resistance, reduced renal plasma flow, increased filtration fraction, lower GFR, and progressive structural and functional deterioration of the kidney. Moreover, both PTC senescence and tubulointerstitial fibrosis are known to contribute to age-associated loss of kidney function. Aging kidneys are also characterized by common hallmarks of cellular senescence, including increased expression of P16 and P21, and increased SA-β-gal activity. Accumulation of the extracellular matrix in the tubulointerstitial space is mediated primarily by myofibroblasts, and EMT serves as a source of interstitial, matrix-producing myofibroblasts [19]. Efforts to understand age-related changes in kidney function, and to elucidate the mechanisms that underlie these changes, may help to focus future research efforts to identify potential therapeutic interventions.

Here, we first examined compared age-related metabolic parameters and renal function between the groups of rats. Compared to the young rats, increases in age-related proteinuria, hypercholesterolemia and hypertriglyceridemia were observed in the older rats (Table 1).

We proceeded to demonstrate increased cellular senescence, as indicated by overexpression of P16, P21 and SA-β-gal in the kidneys of the aging rats. Cellular senescence is not only a marker of renal aging but also actively participates in the process. Furthermore, senescent cells secrete inflammatory factors and growth factors, resulting in a complex shift within the cellular microenvironment, which induces EMT. Increased levels of EMT as a function of age were demonstrated in this study. We found that EMT was increased in the older rats, compared with the younger rats, indicating that EMT increases as a function of age. Given that cellular senescence and EMT contribute to the decline in renal function with age, we investigated the effect of interventions on both senescence and age-related EMT in our rat models. While previous studies have shown that CR decreased the abundance of senescent cells, improved telomere maintenance and reduced the levels of oxidative damage markers in the small intestine and liver [20], and alleviated age-related increase in EMT in the thymus [21], similar studies in the aging kidney have been lacking.

Consistent with previous reports [22–25], short-term CR was found to decrease proteinuria and hypertriglyceridemia, but to have no similar observable effect on hypercholesterolemia. This is, to our knowledge, the first demonstration of the effect of metformin on nondiabetic proteinuria.

Here we report that short-term CR alleviates cellular senescence and EMT in the aging kidney. However, it should be pointed out that even were our study to substantiate short-term CR as an effective intervention for cell senescence and EMT, the degree of restriction required would limit the utility of this intervention. As an alternative strategy, new research has focused on the development of CRM. The objective of CRM research is to identify compounds that mimic the effects of CR by targeting metabolic and stress response pathways affected by CR without actually restricting caloric intake [26]. Previous studies of CRM indicated that metformin reduced the degree of SAHF, the average nuclear area, and the nuclear accumulation of γH2AX in skin fibroblasts. In addition, metformin treatment quantitatively reduced the number of fibroblasts with large nuclear areas, as well as the number of fibroblasts positive for SA-β-gal activity and intense nuclear γH2AX staining [10]. Metformin has also been shown to impede the generation of the cancer stem cell phenotype via transcriptional repression of key drivers of the EMT genetic program, such as ZEB1. Moreover, metformin impeded the TGF-β-promoted loss of the epithelial marker E-cadherin in MCF-7 breast cancer cells and prevented TGF-induced cell scattering and accumulation of the mesenchymal marker vimentin in Madin-Darby canine kidney cells [11, 12]. The present study substantiates the beneficial effect of metformin treatment on cellular senescence and EMT in the aging kidney.

With respect to how short-term CR and metformin treatment might directly impact cellular senescence and EMT, one interesting candidate is the AMPK-mTOR signaling pathway. AMPK is a heterotrimeric serine/threonine protein kinase complex that has been shown to be directly activated by CR and metformin [27, 28], and mTOR has been recognized as a downstream target of AMPK. A series of phosphorylation steps leads to subsequent repression of mTOR activity through cellular energy stress-induced AMPK activation [29, 30]. AMPK has been shown to directly phosphorylate the mTOR binding partner Raptor, and Raptor phosphorylation is known to be required to inhibit mTOR activity under energy-deprived conditions [31]. Moreover, mTOR activation induces senescence in human fibroblasts [32], and Wnt1-driven activation of the mTOR pathway causes senescence and loss of epithelial stem cells after a short hyperproliferative period [33]. Consistent with these observations, we propose that short-term CR and metformin treatment can alleviate cellular senescence and EMT via activation of AMPK/mTOR signaling.

In the *in vivo* experiments, we demonstrated that AMPK/mTOR signaling in kidney was downregulated with age, and that this was reversed by short-term CR and metformin treatment. In order to further verify this pathway, we induced EMT and cellular senescence of PTCs *in vitro* with high glucose [34, 35]. We found that exposure of PTCs to high glucose for 48 h resulted in the high glucose-induced EMT and cellular senescence, decreased expression of activated AMPK and decreased AMPK/mTOR signaling. Costimulation of PTCs with high glucose and resveratrol or metformin, both of which activate AMPK [28, 36], alleviated high glucose-induced EMT and cellular senescence, and increased AMPK/mTOR signaling. Moreover, mTOR was upregulated, and EMT and senescence were increased in AMPK-silenced cells, but were not alleviated in AMPK-silenced cells that had been treated with metformin or resveratrol. These results indicated that metformin and resveratrol inhibited EMT and senescence of PTC via AMPK/mTOR signaling. It is possible that the data presented here could be extrapolated to explain the mechanisms of fibrosis seen in other organs during aging, and to provide strategies to overcome this process.

In conclusion, our study furnishes a mechanistic link between the AMPK-dependent abrogation of senescence by short-term CR or CRM and the development of the age-related profibrotic phenotype. Remarkably, short-term CR or CRM showed simultaneous protective effects on renal senescence and aging-related EMT, further emphasizing the importance of anti-aging treatments in the protection of kidney function in the elderly.

## MATERIALS AND METHODS

### Animals

Male Sprague-Dawley rats were maintained under specific pathogen-free conditions with 22±1°C, 40% humidity, 12:12 h light/dark cycle, one rat per cage, and free access to water. The experiments involving animals included 3-month-old male SD rats (YAL, *n* = 6) and 25-month-old male SD rats (*n* = 24), and all of these experiments were approved by the Institutional Animal Care and Use Committee at the Chinese PLA General Hospital. The 25-month-old male SD rats were divided into 3 groups: OAL, OCR and OMET (*n* = 8 per group). During the following 8 weeks, the OAL group was fed ad libitum, whereas the OCR group was fed an amount of food corresponding to 60% of that ingested by the OAL group over the same time period. Food consumption was measured every 2 weeks. Rats in the OMET group were treated with metformin at 300 mg/kg/d solubilized in water and had free access to food. At the end of the 8 week period, all rats were anesthetized by an intraperitoneal injection of sodium pentobarbital (40 mg/kg). Kidney tissues were removed and perfused with ice-cold, isotonic phosphate-buffered saline (PBS; pH 7.4) to remove any remaining blood. A portion of the kidney tissue was immersed in OCT compound (Tissue-Tek; Sakura Finetek, Torrance, CA, USA) for immunofluorescence staining. The remaining tissue was immediately frozen in liquid nitrogen and stored at −80°C until further processing.

### Cell preparation and culture conditions

Segments of macroscopically and histologically normal renal cortex were obtained aseptically from adult human kidneys that had been surgically removed due to the appearance of small (<6 cm) renal adenocarcinomas, pelviureteric transitional cell carcinomas or benign angiomyolipomas. Informed consent was obtained prior to each operative procedure, and the use of the human renal tissue for primary culture was reviewed and approved by the Human Medical Research Ethics Committee at the Chinese PLA General Hospital. Isolation and incubation of human PTCs were performed as described previously [37, 38]. Briefly, renal cortical tissue was dissected from the medulla, minced, digested with collagenase (class II, 300 U/mg; Sigma Chemical Co., St. Louis, MO, USA) and passed through 100 μm and 200 μm meshes. The filtered cells were resuspended in 45% Percoll (Pharmacia, Peapack, NJ, USA) and separated into four distinct bands by isopycnic centrifugation. The lowest band was removed for culture. PTCs were resuspended in Dulbecco’s modified Eagle’s medium (DMEM)/F12 media (containing 5.5 mM D-glucose; Hyclone, Logan, UT, USA), supplemented with 15% FBS, penicillin (50 U/mL, Sigma Chemical Co), streptomycin (50 μg/mL; Sigma Chemical Co.), human transferrin (5 μg/mL; Sigma Chemical Co.), bovine insulin (5 μg/mL; Sigma Chemical Co.) and EGF (10 ng/mL; Sigma Chemical Co.). The tubular fragments were plated at a density of 1.5 mg pellet/cm2 in 25 cm2 flasks. The cells were incubated in a humidified atmosphere of 95% air/5% CO2 at 37°C (Sanyo Corporation), and the medium was changed every 48 hours. Cells in passages 3 to 5 were selected for this study. In order to explore the effect of high glucose on PTCs, serum-deprived cells were incubated separately in normal glucose control [cultured in 5.5 mM D-glucose (NG)], osmotic control [cultured in 5.5 mM D-glucose plus 24.5 mM mannitol (OS)] and highglucose group [cultured in 30 mM D-glucose (HG)]. In order to explore the effects of metformin and resveratrol on cellular senescence and EMT in PTCs, cells were treated with 2 mmol/L metformin or 50 μmol/L resveratrol.

### Transfection with plasmid

Cells were cultured to 50-60% confluence prior to transfection. Dilute siRNA-AMPK or siRNA-con in 200 μl of jetPRIME™ buffer (jetPRIMETM, Polyplus-transfection, Strasbourg, France) was vortexed and spun down prior to the addition of 8 μl of jetPRIME™ reagent (jetPRIMETM, Polyplus-transfection). The mixture was then vortexed, spun down, and incubated for 10 min at room temperature. Finally, the entire transfection mix was added to 5 mL of medium. The final concentration of the siRNA-AMPK and siRNA-con was 30 nM per well. The medium was changed after 24 h, and the cells were then incubated with 2 mmol/L metformin or 50 μmol/L resveratrol for 24 h.

### Western blotting

Tissues or cells were lysed in 1 mL of 1×RIPA Buffer containing 1 μL leupeptin (Amresco LLC, Solon, OH, USA), 1 μL aprotinin (Amresco), and 10 μL phenylmethylsufonyl fluoride (Amresco). A total of 60-100 μg of the extracted proteins was separated by 6-12% SDS-polyacrylamide gel electrophoresis (Bio-Rad, Hercules, CA, USA) and electrotransferred (DYCP-40C; Liuyi Instrument Factory, Beijing, China) onto nitrocellulose membranes (Merck Millipore, Billerica, MA, USA). The membranes were blocked with casein for 1 h at room temperature and subsequently incubated with the following primary antibodies at 4°C overnight: anti-E-cadherin (Rabbit; Abcam, Cambridge, MA, USA) at 1:1000, anti-a-SMA (Mouse; Santa Cruz Biotechnology, Dallas, TX, USA) at 1:500, anti-Zeb1 (Rabbit; Abcam) at 1:500, anti-AMPK1 (Rabbit; Abcam) at 1:500, anti-AMPK alpha1+AMPK alpha2 (Rabbit; phosphoS485+S491, Abcam) at 1:500, anti-P16 (Mouse; Abcam) at 1:1000, anti-P21 (Rabbit; Cell Signaling, Boston, MA, USA) at 1:5000, anti-mTOR (Rabbit; Abcam) at 1:500, or anti-phospho-mTOR (Rabbit; Abcam) at 1:500. After being washed with Tris-buffered saline containing 0.1% Tween (TBST) 20, the membranes were probed with horseradish peroxidase-conjugated anti-mouse or anti-rabbit IgG (1:1000 dilution; Beyotime Institute of Biotechnology, Shanghai, China). After washing again with TBST, the bands were visualized using an enhanced chemiluminescence system (DP2-BSW; Olympus, Tokyo, Japan) and densitometry was performed using ImageJ (Wayne Rasband, National Institutes of Health, Bethesda, MD, USA).

### Immunofluorescent staining

PTCs were seeded into wells and cultured for 24 h until the cells reached confluence. After stimulation, the cells were fixed in 4% paraformaldehyde for 15 min at 4°C; permeabilized with 0.1% Triton X-100; blocked with 5% bovine serum albumin for 15 min at room temperature; and incubated simultaneously with anti-E-cadherin (1:100) and anti-α-SMA (1:50) at 4°C overnight. Then, the cells were probed with fluorescein isothiocyanate-conjugated anti-mouse IgG (1:100; Beyotime Institute of Biotechnology) and CY3-conjugated anti-rabbit IgG (1:400; Beyotime Institute of Biotechnology) for 1.5 h in dark. Finally, the cells were incubated with Mounting Medium with DAPI (Zhongshan Golden bridge Biotechnology, Beijing, China) in the dark for 5 min. The immunoassayed proteins were observed using a fluorescence microscope (FV10-ASW; Olympus), and the cells were photographed using a confocal microscope (Radiance 2000; Bio-Rad). Renal tissues were embedded in OCT compound. Cryostat sections (4 μm) were fixed in 4% paraformaldehyde and blocked with casein, and then stained directly with anti-E-cadherin and anti-α-SMA. The subsequent procedures were the same as described above.

### Senescence-associated β-galactosidase (SA-β-gal) staining

Cryostat sections (4 μm) were fixed in 0.2% glutaraldehyde and 2% formaldehyde at room temperature for 15 min, and then washed in PBS and incubated in freshly prepared SA-β-gal staining solution (1 mg/mL X-gal, 40 mM citric acid/sodium phosphate (pH 6.0), 5 mM potassium ferrocyanide, 150 mM NaCl, and 2 mM MgCl2) at 37°C without CO2 overnight. The tissue sections were counterstained with eosin and examined under a microscope.

### Senescence-associated focal heterochromatin (SAFH) analysis

PTCs were cultured directly on glass cover slips and then fixed with 4% paraformaldehyde and permeabilized with 0.1% Triton X-100/PBS for 10 min. The DNA was visualized following staining with DAPI (1 mg/mL) for 3 min. The cover slips were mounted in a 90% glycerol/PBS solution and examined using a laser confocal microscope.

### Statistical analysis

All experiments were repeated at least three times. The results are expressed as the means ± SD. Statistical analyses were performed using analysis of variance with SPSS software, version 17.0 (SPSS Inc., Chicago, IL, USA), and a level of *p* < 0.05 was considered statistically significant.

## Abbreviations

CR: caloric restriction
PTC: primary proximal tubular cells
CRMs: caloric restriction mimetics
CKD: chronic kidney disease
SA-β-gal: Senescence-associated β-galactosidase
SAFH: Senescence-associated focal heterochromatin
EMT: epithelial-mesenchymal transition

## References

[1] Xue JL, Ma JZ, Louis TA, Collins AJ (2001). Forecast of the number of patients with end-stage renal disease in the United States to the year 2010. Journal of the American Society of Nephrology.

[2] Coresh J, Astor BC, Greene T, Eknoyan G, Levey AS (2003). Prevalence of chronic kidney disease and decreased kidney function in the adult US population: Third National Health and Nutrition Examination Survey. American journal of kidney diseases.

[3] Anderson S, Halter JB, Hazzard WR, Himmelfarb J, Horne FM, Kaysen GA, Kusek JW, Nayfield SG, Schmader K, Tian Y, Ashworth JR, Clayton CP, Parker RP, Tarver ED, Woolard NF, High KP (2009). Prediction, progression, and outcomes of chronic kidney disease in older adults. J Am Soc Nephrol.

[4] Campisi J, d’Adda di Fagagna F (2007). Cellular senescence: when bad things happen to good cells. Nature reviews Molecular cell biology.

[5] Evan GI, d’Adda di Fagagna F (2009). Cellular senescence: hot or what?. Current opinion in genetics & development.

[6] Zhou XJ, Rakheja D, Yu X, Saxena R, Vaziri ND, Silva FG (2008). The aging kidney. Kidney international.

[7] Liu Y (2009). New Insights into Epithelial-Mesenchymal Transition in Kidney Fibrosis. Journal of the American Society of Nephrology.

[8] Schmitt R, Cantley LG (2008). The impact of aging on kidney repair. Am J Physiol Renal Physiol.

[9] Ning YC, Cai GY, Zhuo L, Gao JJ, Dong D, Cui S, Feng Z, Shi SZ, Bai XY, Sun XF, Chen XM (2013). Short-term calorie restriction protects against renal senescence of aged rats by increasing autophagic activity and reducing oxidative damage. Mechanisms of ageing and development.

[10] Arkad’eva AV, Mamonov AA, Popovich IG, Anisimov VN, Mikhel’son VM, Spivak IM (2011). [Metformin slows down ageing processes at the cellular level in SHR mice]. Tsitologiia.

[11] Cufi S, Vazquez-Martin A, Oliveras-Ferraros C, Martin-Castillo B, Joven J, Menendez JA (2010). Metformin against TGFbeta-induced epithelial-to-mesenchymal transition (EMT): from cancer stem cells to aging-associated fibrosis. Cell Cycle.

[12] Vazquez-Martin A, Oliveras-Ferraros C, Cufí S, S Del Barco, Martin-Castillo B, Menendez JA (2010). Metformin regulates breast cancer stem cell Ontogeny by transcriptional regulation of the Epithelial-Mesenchymal Transition (EMT) status. Cell Cycle.

[13] Xia L, Wang XX, Hu XS, Guo XG, Shang YP, Chen HJ, Zeng CL, Zhang FR, Chen JZ (2008). Resveratrol reduces endothelial progenitor cells senescence through augmentation of telomerase activity by Akt-dependent mechanisms. Br J Pharmacol.

[14] Demidenko ZN, Blagosklonny MV (2009). At concentrations that inhibit mTOR, resveratrol suppresses cellular senescence. Cell Cycle.

[15] Vergara D, Valente CM, Tinelli A, Siciliano C, Lorusso V, Acierno R, Giovinazzo G, Santino A, Storelli C, Maffia M (2011). Resveratrol inhibits the epidermal growth factor-induced epithelial mesenchymal transition in MCF-7 cells. Cancer letters.

[16] Peinado H, Olmeda D, Snail Cano A (2007). Zeb and bHLH factors in tumour progression: an alliance against the epithelial phenotype?. Nature Reviews Cancer.

[17] Blagosklonny MV (2007). An anti-aging drug today: from senescence-promoting genes to anti-aging pill. Drug Discov Today.

[18] Dimri GP, Lee X, Basile G, Acosta M, Scott G, Roskelley C, Medrano EE, Linskens M, Rubelj I, Pereira-Smith O (1995). A biomarker that identifies senescent human cells in culture and in aging skin in vivo. Proc Natl Acad Sci U S A.

[19] Liu Y (2004). Epithelial to mesenchymal transition in renal fibrogenesis: pathologic significance, molecular mechanism, and therapeutic intervention. Journal of the American Society of Nephrology.

[20] Wang C, Maddick M, Miwa S, Jurk D, Czapiewski R, Saretzki G, Langie SA, Godschalk RW, Cameron K, von Zglinicki T (2010). Adult-onset, short-term dietary restriction reduces cell senescence in mice. Aging (Albany NY).

[21] Yang H, Youm YH, Dixit VD (2009). Inhibition of Thymic Adipogenesis by Caloric Restriction Is Coupled with Reduction in Age-Related Thymic Involution. The Journal of Immunology.

[22] Cui J, Bai XY, Shi S, Cui S, Hong Q, Cai G, Chen X (2011). Age-related changes in the function of autophagy in rat kidneys. Age (Dordr).

[23] Everitt AV, Porter BD, Wyndham JR (1982). Effects of caloric intake and dietary composition on the development of proteinuria, age-associated renal disease and longevity in the male rat. Gerontology.

[24] Reaven GM, Reaven EP (1981). Prevention of age-related hypertriglyceridemia by caloric restriction and exercise training in the rat. Metabolism: clinical and experimental.

[25] Anisimov VN, Berstein LM, Egormin PA, Piskunova TS, Popovich IG, Zabezhinski MA, Tyndyk ML, Yurova MV, Kovalenko IG, Poroshina TE, Semenchenko AV (2008). Metformin slows down aging and extends life span of female SHR mice. Cell Cycle.

[26] Ingram DK, Zhu M, Mamczarz J, Zou S, Lane MA, Roth GS, deCabo R (2006). Calorie restriction mimetics: an emerging research field. Aging cell.

[27] Zhou G, Myers R, Li Y, Chen Y, Shen X, Fenyk-Melody J, Wu M, Ventre J, Doebber T, Fujii N, Musi N, Hirshman MF, Goodyear LJ, Moller DE (2001). Role of AMP-activated protein kinase in mechanism of metformin action. The Journal of clinical investigation.

[28] Mouchiroud L, Molin L, Dalliere N, Solari F (2010). Life span extension by resveratrol, rapamycin, and metformin: The promise of dietary restriction mimetics for an healthy aging. Biofactors.

[29] Hardie DG (2004). The AMP-activated protein kinase pathway—new players upstream and downstream. J Cell Sci.

[30] Inoki K, Ouyang H, Zhu T, Lindvall C, Wang Y, Zhang X, Yang Q, Bennett C, Harada Y, Stankunas K, Wang C-y, He X, MacDougald OA, You M, Williams BO, Guan K-L (2006). TSC2 Integrates Wnt and Energy Signals via a Coordinated Phosphorylation by AMPK and GSK3 to Regulate Cell Growth. Cell.

[31] Gwinn DM, Shackelford DB, Egan DF, Mihaylova MM, Mery A, Vasquez DS, Turk BE, Shaw RJ (2008). AMPK Phosphorylation of Raptor Mediates a Metabolic Checkpoint. Molecular Cell.

[32] Zhuo L, Cai G, Liu F, Fu B, Liu W, Hong Q, Ma Q, Peng Y, Wang J, Chen X (2009). Expression and mechanism of mammalian target of rapamycin in age-related renal cell senescence and organ aging. Mechanisms of ageing and development.

[33] Castilho RM, Squarize CH, Chodosh LA, Williams BO, Gutkind JS (2009). mTOR mediates Wnt-induced epidermal stem cell exhaustion and aging. Cell Stem Cell.

[34] YJLaHJ. Han (2009). Troglitazone ameliorates high glucose-induced EMT and dysfunction of SGLTs through PI3K/Akt, GSK-3, Snail1, and -catenin in renal proximal tubule cells. Am J Physiol Renal Physiol.

[35] Cao D, Zhang M, Jiang C, Xue L, Sun C (2011). Protection of Tanshinone IIA to Human Peritoneal Mesothelial Cells (HPMC) through Delaying Cellular Senescence Induced by High Glucose. Renal Failure.

[36] Baur JA, Pearson KJ, Price NL, Jamieson HA, Lerin C, Kalra A, Prabhu VV, Allard JS, Lopez-Lluch G, Lewis K, Pistell PJ, Poosala S, Becker KG, Boss O, Gwinn D, Wang M (2006). Resveratrol improves health and survival of mice on a high-calorie diet. Nature.

[37] Qi W, Johnson DW, Vesey DA, Pollock CA, Chen X (2007). Isolation, propagation and characterization of primary tubule cell culture from human kidney. Nephrology (Carlton).

[38] Vesey DA, Cheung CW, Kruger WA, Poronnik P, Gobe G, Johnson DW (2005). Thrombin stimulates proinflammatory and proliferative responses in primary cultures of human proximal tubule cells. Kidney international.

